# Abstracts: International Symposium on Immunopathogenesis of Chlamydia Infections

**DOI:** 10.1155/S1064744996000385

**Published:** 1996


Infectious Diseases in Obstetrics and Gynecology 4:194–210 (1996)
© 1996 Wiley-Liss, Inc.

### International Symposium on Immunopathogenesis of Chlamydia Infections
### December 6-8, 1996
### Cornell University Medical College
### 1300 York Avenue, New York, NY
### USA

### Abstracts

Abstracts

Received September 15, 1996
Accepted October 1, 1996

cytokines in *C. pneumoniae* infection.

### Cytokine Profile Of *Chlamydia Pneumoniae* Infected U-937 Macrophages

**[1]CA Gaydos, [1]DG Pham, [1]L Bono, [2]TC Quinn.**

*[1]Johns Hopkins University, Baltimore, MD, [2]NIH, NIAID, Bethesda, MD*

| Time | Titer | IL-1β | | IFNγ | | TNF-α | |
|---|---|---|---|---|---|---|---|
| | IFU/ml | Control | Infected | Control | Infected | Control | Infected |
| 24 hr | $6.3 \times 10^3$ | 15.71 | 13.96 | 16.06 | 9.45 | 134.18 | 110.22 |
| 48 hr | $1.7 \times 10^3$ | 8.88 | 8.88 | 4.68 | 5.05 | 72.56 | 79.41 |
| 72 hr | $1.4 \times 10^4$ | 27.23 | 1.29 | 1.29 | 19.64 | 131.43 | 247.82 |
| 96 hr | $3.6 \times 10^3$ | 48.34 | BLD[a] | BLD | 48.08 | 365.56 | 447.71 |
| 7 d | 0 | 46.23 | BLD | BLD | BLD | 202.51 | 421.59 |

[a] BLD= Below the level of detection. Control growth media was BLD for all 3 assays.

It has been demonstrated that *C. pneumoniae* can infect U-937 human macrophages and it has been postulated that alveolar macrophages may play a role in the initial pulmonary infectious process. Since it has been shown that *C. trachomatis* can induce mediators of inflammation and persistence and in order to begin to understand the pathogenesis of infections due to *C. pneumoniae*, we have investigated the cytokine response of U-937 cells infected *in vitro* with *C. pneumoniae*, strain 2023. Non-adherent U-937 macrophages (4 ml of $10^6$/ml) were infected with 1 ml of 3.5 x $10^4$IFU/ml of *C. pneumoniae* by gentle agitation every 20 min. for 6 hr, after which, 10 ml of growth media without cyclohexamide was added. Flasks were sampled by removing and freezing 1 ml of the infected cells at 24 hr., 48 hr, and 7 days. A control flask was treated in an identical manner, except that uninfected growth media was used in place of the inoculum. After the 48, 72, and 96 hr. samples, the flasks were split 1:2, in order to maintain persistence and viability. Titers of *C. pneumoniae* were determined for each sample time point from the infected flask in Hep-2 cells. Cytokine levels were determined from the samples from both the infected and non-infected flasks for the following: IL-1β, IFNγ, and TNF-α. The assays were performed by sandwich ELISA using reagents from Endogen™. Both titers and cytokine levels were adjusted to reflect the dilution due to splitting of the cells in the flasks. The results of the viability titers and cytokine levels (pg/ml) are shown in the table.

These results indicate that *C. pneumoniae* infection in U-937 macrophages stimulate increased production of IL-1β by 96 hr. and IFNγ as well as TNF-α by 72 hr. Mechanisms of action of these cytokines may include, but are not limited to, induction of adhesion molecule expression on endothelial and epithelial cells (IL-1β); enhancement of intracellular killing, TNF cytotoxicity, and NK cell activity (IFNγ); and enhanced macrophage killing and cytotoxic T cell differentiation (TNF-α). Further study will enhance our understanding of the role of these and other

### Production Of Il-4 And IL-6 In HEp-2 Cells Infected With *Chlamydia pneumoniae*

***PM Roblin, A Kutlin, MR Hammerschlag***
*Department of Pediatrics, SUNY Health Science Center @ Brooklyn, NY*

**Introduction:** An association of *Chlamydia pneumoniae* infection and reactive airway disease has been shown in children. Anti- *C. pneumoniae* IgE was demonstrated by immunoblot in 85.7% of culture positive asthmatic children with wheezing compared with only 9.1% culture positive children with pneumonia. Cytokines initiate and perpetuate the chronic inflammaation of asthma. IL-4 plays a critical role in switching of B lymphocytes to produce IgE, and may therefore be of critical importance in the development of atopy. IL-6 can act synergistically with IL-4. In this study we measured the production of IL-4 and IL-6 from *C. pneumoniae* infected HEp-2 at various time points.

**Methods:** Confluent monolayers of HEp-2 cells were inoculated with seven *C. pneumoniae* isolates: J21, AR39, BAL16, CDC8, JCW480, BAL15, TW1183, at 104 IFU/ml in cycloheximide free media. All results were calculated against moc-infected HEp-2 cell assayed in a sandwich ELISA for IL-4 (Intertest-IL4, Genzyme Corp., MA) and IL-6 (Biokine IL-6, T Cell Diagnostics, Inc., MA).

**Results:** The results are shown below:

| *C. pneumoniae* isolates | IL-6 (pg/ml) | | | IL-4 (ng/ml) | | |
|---|---|---|---|---|---|---|
| | 24hr | 48hr | 72hr | 24hr | 48hr | 72hr |
| J21 | 33.69 | 39.57 | 58.55 | 0 | 0 | 0.016 |
| AR39 | 28.53 | 41.63 | 45.15 | 0 | 0 | 0.26 |
| BAL16 | 14.71 | 32.35 | 38.24 | 0 | 0 | 0 |
| CDC8 | 26.47 | 11.76 | 8.83 | 0 | 0 | 0 |
| JCW480 | 8.82 | 26.47 | 38.24 | 0 | 0 | 0.082 |
| BAL15 | 26.47 | 5.88 | 47.21 | 0 | 0 | 0 |
| TW183 | 11.76 | 17.65 | 41.18 | 0 | 0 | 0 |

There was little or no detectable production of IL-4. We also assayed for production of IL-4 and IL-6 in a HEp-2 cell line chronically infected with TW183. These cells have been maintained for approximately 1 year without centrifugation, cycloheximide or addition of fresh cells. Supernatants were assayed from these infected cells for IL-4 and IL-6 at a "lysis" stage where a majority of cells appear lysed and a "recovery" stage where remaining cells re-seed and form a monolayer, at this stage cells are split into fresh flasks. IL-4 production was 10 times greater than that seen with "acutely" infected monolayers (0.135 ng/ml). IL-6 was 100 times higher at lysis stage, 2,044.0 pg/ml and 10 times greater at the recovery stage, 352.8 pg/ml.

**Conclusions:** *C. pneumoniae* stimulated the production of IL-6 and minimally stimulated the production of IL-4 in HEp-2 cells.

### *Chlamydia pneumoniae* Induced Atherosclerosis In Rabbits

***K Laitinen, P Saikku.***
*National Public Health Institute, Oulu, Finland*

*Chlamydia pneumoniae* (Cpn), an obligatory intracellular, Gram-negative bacterium, is a common cause of respiratory tract infections worldwide. In addition to acute infections it has been associated with chronic lung processes. The most important finding is evidently the association of chronic Cpn infection with cardiovascular diseases. The first serological association was published in 1988 in Finland, and after that several groups worldwide have found the association between CHD and Cpn antibodies. Direct evidence between Cpn and CHD is demonstrated by finding Cpn organisms in atherosclerotic lesions. It is also shown that Cpn can infect human endothelial cells and macrophages in vitro, and according to recent evidence these cells could carry Cpn into the smooth muscle cells below the lining of the artery. Even if the association between Cpn infection and atherosclerosis has been confirmed using several different methodological approaches by several laboratories around the world, the causal relationship between chronic Cpn infection and the development of atherosclerosis can be indicated only by animal experiments and intervention trials. Mouse has already proved to be useful animal for the study of atherosclerosis, and the susceptibility of artherosclerosis varies greatly between mouse strains. With advances in molecular biology mouse strains with genetic defects or overexpression of certain genes involved in lipoprotein metabolism have been created and are of great value for studies in atherogenesis. Mice are also susceptible to Cpn infection and develop latent chlamydial lung infection. Another animal model to study artherosclerosis is the New Zealand White (NZW) rabbit on a high cholesterol diet, and hereditary hyperlipemic Watanabe rabbit (WHHL). Moazed et al. has recently shown that Cpn causes a moderate, self-limiting interstitial pneumonia in rabbits, and the infection mimics the human infection. Purpose of our study was to determine whether this rabbit model is suitable for studying the development of atherosclerosis after Cpn infection. Animals used were Bordetella bronchiseptica and Pasteurella spp. free male New Zealand White rabbits (5 mo old). Rabbits were inoculated intranasally with a total volume of 0.5 ml of organisms (2x10 7 IFU/ml Cpn strain K7). Control animals were inoculated with 0.5 ml sterile SPG medium. Rabbits were reinfected in the same manner and with the same dose 3 weeks after primary inoculation. Rabbits were observed daily for signs of disease and weighed twice a week. Samples were collected 2 weeks after primary infection and 1, 2 and 4 weeks after reinfection (5 and 1 control/each timepoint). Lung, spleen and liver tissues were homogenized. Isolation was done in HL cell culture and inclusions were detected with FITC-conjugated chlamydia genus-specific antibodies. Lung. spleen, liver, heart and ascending aorta were fixed in 10% buffered formalin immediately after removal for histology and immunohistochemistry. No clinical signs of disease were observed except in two animals 24 and 48 hrs after reinfection. One animal developed severe lung oedema and the other animal had acute pneumonia. All sample homogenates (lung, liver, spleen) remained culture negative during the course of the infection. Five weeks after primary infection two out of four rabbits developed early signs of fibrous plaques in aortas and seven weeks after primary infection three rabbits (3/5) developed plaques in ascending aortas. These animals were also found positive in immunohistochemistry. Control animals had no signs of atherosclerotic changes.

### SeroCP - A Serological Assay For The Detection Of *Chlamydia pneumoniae* IgG, IgA and IgM Antibodies

***A Groh, V Prankratova, M Lipson*, A Ben-Michael*, O Horovitz, and R Mosckovitz*.***
*Institute of Medical Microbiology, Friedrich Schiller University of Jena, Germany, *Department of Research and Development, Savyon Diagnostics, Kiryat Minrav, Ashdod, 77101 Israel*

**Objective:** *Chlamydia pneumoniae* is a widely spread pathogen responsible for infections of the upper and lower respiratory tracts. Specific antibody prevalence of *C. pneumoniae* is over 50% among adults worldwide, and is low in pre-school children. Recently, there has been a growing number of reports which suggest an association between chronic *C. pneumoniae* infections and coronary artery disease. Additional studies stress the role of this pathogen in Adult Onset Asthma. The SeroCP has been developed for the determination of *C. pneumoniae* IgG, IgA, and IgM antibodies.

**Materials and Methods:** SeroCP uses as antigen *C. pneumoniae* strain TWAR 183 purified elementary bodies (EBs). Serum samples were obtained from 13 adult patients positive for C. peumoniae by PCR (PCRP), 28 patients positive for *C. pneumoniae* by MIF-IgG ≥1:512(CWP), 35 children negative for C. preumoniae and *C. trachomatis* by MIF(CNPNT) and 49 healthy adults(HA). The *C. pneumoniae* MIF were interpreted as positive for IgG ≥1:32, for IgA ≥1:20 ad for IgM ≥1:20.

**Results:** The prevalence (%) of *C. pneumoniae* antibodies in different patient groups as detected by MIF and SeroCP, is summarized in the following table:

| | GROUP | No. of Patients | SeroCP(%) | MIF(%) |
|---|---|---|---|---|
| IgG | PCRP | 13 | 85 | 92 |
| | CWP | 28 | 100 | 100 |
| | CNPNT | 35 | 3 | 0 |
| | HA | 49 | 76 | 98 |
| IgA | PCRP | 13 | 92 | 54 |
| | CWP | 28 | 100 | 96 |
| | CNPNT | 35 | 6 | 0 |
| | HA | 49 | 40 | 41 |
| IgM | PCRP | 13 | 0 | 0 |
| | CWP | 28 | 21 | 0 |
| | CNPNT | 35 | 0 | 0 |
| | HA | 49 | 2 | 0 |

**Conclusions:** SeroCP is highly sensitive and specific and well correlated with PCR and MIF. The MIF IgG titer 1:64 correlates with positive SeroCP IgG and MIF IgA titer 1:20 with SeroCP IgA. The ELISA based SeroCP is easy and convenient to perform as compared to the technically demanding MIF test. In addition to IgG and IgA, SeroCP IgM testing should be recommended for the adequate and sensitive diagnosis, when IgG and IgA antibodies are produced in low levels.

### Protective Immunity Against *Chlamydia psittaci* Serotype I Strains

***[1]DS Carlos, [2]S Jesus, [1]B Francoise, [1]S Armel, [1]R Annie.***

*[1]Pathologie Infectieuse et Immunologie, INRA, [3]7380 Nouzilly, France, [2]Dept. Patologia Animal, Facultad Veterinaria, Campus de Espinardo, Murcia, Spain*

The serotype 1 stains of *Chlamydia psittaci* induce several diseases among ruminants. One of them, enzootic abortion of ewes, causes significant economic losses for farmers and represents a danger for pregnant women. This disease results from the colonization of the placenta by *Chlamydia*. Transmission of the infection, in a flock or to humans, results mainly from the massive excretion of *Chlamydia* which occurs at lambing or abortion, in infected placentas and uterine fluids.

The immunity following abortion of ewes is strong enough to prevent abortion and chlamydial shedding during the next pregnancy. Killed vaccines, used in the past, reduced the incidence of abortions but could not prevent excretion. We developed a thermosensitive strain of *C. psittaci* (1) which provided a live vaccine against abortive chlamydiosis. This vaccine is able to avoid abortion and chlamydial excretion at lambing. However, the use of a recombinant protective antigen will be safer. This new vaccine should be as efficient as the live vaccine and should allow the specific diagnosis of abortive strains in vaccinated flock. In order to detect potential protective antigens, the cellular and humoral immunity against *C. psittaci* abortive strains was studied in a mouse model of systemic infection (2).

Donors mice were intravenously infected with live *C. psittaci*. One month after inoculation splenic cells were transferred to naive recipient mice, which were challenged with $2.10^5$ plaque forming units (pfu) of a virulent strain one day later. Protection was assessed after 6 days by measuring the decrease in spleen infectivity. This model showed that lyt-$2^+$ T cells play the major role in the protection (3)

The importance of humoral immunity was assessed in a pregnant mouse model. Mice were passively immunized intravenously at day 11 of gestation with polyclonal sera or monoclonal antibodies (mabs). Mice were challenged on the following day, and protection was measured by placental and fetal colonization four days later. The number of living offspring 8 days after birth was also used to assess the protection. Results showed that immune sera and type specific mabs could transfer protection, probably by neutralizing chlamydiae during the bacteremia which proceeds placental colonization.

Therefore, mabs raised against the ovine abortive strain AB7 were produced in order to detect molecules bearing potential protective epitopes. The protective effect of these mabs was tested *in vitro* by neutralization assays. The mabs selected *in vitro* were tested for their ability to protect pregnant mice after passive immunization. All the protective mabs were directed against heat sensitive epitopes located on an 110 kDa (apparent molecular weight under non denaturing conditions) oligomer of the Major Outer Membrane Protein (MOMP) of *C. psittaci* strain AB7 (4). The protective mabs did not react with the 39 kDa monomeric MOMP which is produced after heat denaturation.

Protection experiments were performed on a pregnant mouse model with vaccines containing mainly native oligometric MOMP. Different formulations were tested and mice were immunized with two injections given at 15-days interval. They were challenged intraperitioneally at day 11 of pregnancy (1 month after the last vaccination) with $2.10^5$ pfu of the AB7 ovine abortion strain. Protection was evaluated by the number of living offspring per litter and by the level of infection in the livers, fetus and placentas (5). The level of infection in the placentas was the most discriminant test.

Mice vaccinated with non-denatured MOMP extracts were better protected than mice which have received a heat denatured MOMP extract. The infection of the placenta was significantly higher for mice which have received the heat denatured vaccine, whereas the difference seen in the number of living offspring between the 2 groups, was less significative. Mice vaccinated with the heat denatured vaccine were not protected from placental infection but they were partially protected from abortion. As previous works have shown that mabs raised against monomeric denatured MOMP were not protective, this partial protection could be the result of T cell epitopes located on MOMP.

In definitive, the good level of protection which have been obtained could be the result of the combination of MOMP native B cell epitopes and MOMP T cell epitopes. In this model, we demonstrated that placental colonization of pregnant mice could be prevented with native oligomeric MOMP. Mice vaccinated with native oligomeric MOMP were protected from abortion but also from infection. The last point is very important since chlamydial excretion at lambing is mainly responsible of the contamination of other animals.

**References**

1. A. Rodolakis: In vitro and in vivo propertics of chemically induced temperature sensitive mutants of *Chlamydia psittaci* var. ovis screening in a murine model. Infect. Immun. 42:525-530, 1983.
2. D. Buzoni-Gatel, A. Rodolakis, M. Plommet: T cell mediated and humoral immunity in a mouse *Chlamydia psittaci* systemic infection. Res. Vet. Sci. 43:59-63, 1987.
3. D. Buzoni-Gatel, L. Guilloteau, F. Bernard, S. Bernard, T. Chardes, A. Rocca: Protection against *Chlamydia psittaci* in mice conferred by Lyt-2+ T cells. Immunol. 77:284-288, 1992.
4. C. De Se, A. Souriau, F. Bernard, J. Salinas, A. Rodolakis: An oligomer of the major outer membrane protein of Chlamydia psittaci is recognized by monoclonal antibodies which protect mice from abortion. Infect. Immun. 63:4912-4916, 1995.
5. C. De Sa, F. Bernard, J. Sallinas, A. Souriau, A. Rodolakis: *Chlamydia psittaci* infection could be prevented in pregnant mice with a vaccine containing native oligomeric major outer membrane protein, submitted.

### Scarring Trachoma Is Associated With Polymorphism In The TNF-Alpha Gene Promoter And With Elevated Tnf-Alpha In Tear Fluid

***DJ Conway, MJ Holland, RL Bailey, AE Campbell, S Mahdi, R Jennings, E Mbena, DCW Mabey.***

*Department of Clinical Sciences, London School of Hygiene & Tropical Medicine, Keppel Street, London WC1E 7HT, UK*

Polymorphisms in the promoter region of the tumor necrosis factor (TNF) -alpha gene have recently been shown to be associated with an increased risk of severe malaria and of mucocutaneous leishmaniasis. In an attempt to determine the role of TNF-alpha in the fibrotic sequelae of human *Chlamydia trachomatis* infection, we have looked for associations between TNF-alpha levels in ocular secretions, and between polymorphisms in the TNF-alpha gene promoter region, and scarring trachoma in a case-control study comparing 153 age, sex and village matched controls in a trachoma endemic Gambian population. A higher proportion of cases (28%) than controls (18%) had the -308A allele, particularly as homozygotes ($X^2$ for trend, p=.032). The trend was similar, but non-significant for the rarer -238A allele, and highly significant for the number of either -308A or 1238A sites in an individual (p=0.003). These associations were independent of HLA class I or class II types. TNF-alpha was detected more frequently in tear samples from cases (38%) than from controls (16%), the association increasing for higher concentrations of TNF-alpha (p=0.015). Among cases, detectable TNF-alpha in tears was highly associated with the presence of ocular chlamydial infection (p<0.001). These results suggest that TNF-alpha plays a major role in the cicatricial sequelae of *C. trachomatis* infection in humans.

### Characterization Of The Local Immune Response To *Chlamydia Trachomatis* Among Trachoma Patients With Conjunctival Scarring

***[2]S Phan, [3]R Pham, [2]D Dean***

*[1]Dept. of Medicine, Division of Infectious Diseases, and the [2]Francis I. Proctor Foundation, University of California at San Francisco School of Medicine, San Francisco, California, and [3]Santa Clara Hospital, San Jose, California*

*Chlamydia trachomatis* is the etiologic agent of trachoma which has a worldwide distribution and is the leading cause of preventable blindness in the world. Progression of disease from repeated infection in childhood is common and results in the sequelae of conjunctival scarring that indirectly leads to blindness. The immunopathogenic mechanisms for the development of scarring are not well defined. In order to address this issue, we evaluated Vietnamese trachoma patients with conjunctival scarring who were undergoing corrective lid surgery for trichiasis. Control were Vietnamese individuals with conjunctival scarring due to other causes. Patients were examined and scored for trachoma based on the World Health Organization trachoma grading scale. Serum was obtained for microimmunofluorscence testing (MIF). Conjunctival swabs were collected for DFA and PCR. Conjunctival biopsies were obtained at the time of surgery for RT-PCR to quantitate and type cytokines and to detect *C. trachomatis*, and for immunohistochemical studies. All cases revealed a grading C3 (severe scarring with distortion of the lid). Five (36%) of 14 cases had documented chlamydial organisms by DFA and/or PCR or RT-PCR; none of the controls were positive for chlamydia. Immunohistochemical studies revealed a statistically significant higher level of CD4 (p<0.04) and CD8 cells (p<0.01), and macrophages (p<0.05) in cases than controls; there were no difference for B cells. Five cases had elevated interferon gamma (IFNγ) levels; these 5 correlated with both a lack of demonstrable organisms by DFA, PCR, or RT-PCR, and elevated IL-2 levels (p<0.034). IL-6, and IL-12 levels were elevated among cases but not controls (p<0.04); only 5 cases had elevated levels for TNFa. There were no differences for IL-4. The data reveal that chlamydia are present in trachoma cases even with burned out disease although viability could only be confirmed in one patient. Elevated IFNγ levels may have contributed to elimination of the organism and lack of identification of *C. trachomatis* in five cases. The cytokine profiles in this study were characteristic of both Th-1 and Th-2 responses, and suggest that these may be important in regulating host immune defenses against persistent *C. trachomatis* infections or persistent chlamydial antigen. Further study is required of the cell mediated immune response to chlamydia which will likely impact the direction of vaccine development of tr

---

### The Relationship Between The 60 Kd Heat Shock Protein (HSP60), Antisperm Antibodies And Undetected *Chlamydia trachomatis* Infection In Male Partners Of Couples With Undiagnosed Infertility

**[1]MG Munoz, [2]J Jeremias, [2]SS Witkin**

*[1]Universidad Simon Bolivar, Caracas, Venezuela and [2]Cornell University Medical College, New York, New York, USA*

**Introduction:** Heat shock protein 60 kD (HSP60) is produced by mammalian cells in response to several stresses, i.e. infection, inflammation and elevated temperature. The aim of this investigation was to demonstrate the relationship between heat shock protein concentration in semen, antisperm antibodies and *Chlamydia trachomatis* infection in the male genital tract.

**Materials and methods:** We examined the presence of HSP60 in semen from 64 male partners of infertile couples with no history of *C. trachomatis* infection and whether its presence was related to sperm antibodies (detected by immunobead binding) and anti-chlamydial antibodies (SERO ELISA, Savyon). HSP60 was detected by an enzyme-linked immunosorbent assay (ELISA) using a monoclonal antibody to HSP60 bound to wells of a microtitre plate and a polyclonal antibody to HSP60 as the detecting antibody (Stress Gen). HSP60-specific mRNA was detected in mononuclear cells isolated from semen by a PCR ELISA.

**Results:** HSP60 was detected in semen from nine (14.1%) men, while HSP60 mRNA was present in 16 (25.0%) samples. Spermatozoa from 12 (18.8%) men had bound auto-antibodies, while 20 (31.3%) semen samples had anti-chlamydial antibodies. The presence of HSP60 in semen was correlated with the occurrence of sperm antibodies ($P= 0.008$), anti-chlamydial immunoglobulin A antibodies ($P= 0.002$) and HSP60-specific mRNA in seminal mononuclear cells ($P= 0.03$).

**Conclusions**: The results demonstrate that HSP60 is produced in the male genital tract and is present in a soluble form in semen in association with genital tract immune activation and/or a genital exposure to *C. trachomatis*. Lymphocyte activation apparently represents one source of HSP60. Non-lymphoid cells or microorganisms may also contribute to the total HSP60 level in semen. The role of HSP60 in activating or inhibiting immune responses within the male genital tract will be the purpose of future studies.

---

### *Chlamydia trachomatis* Specific Immunoparameters And Cytokine Production In Semen From Males Affected By Prostatitis

**F Meacci, A Salvi and S Mazzoli**
*S.T.D. Center, Infectious Disease Unit, Santa Marie Annunziata Hospital, Florence, Italy*

**Introduction**: The etiopathogenetic role of *Chlamydia trachomatis (C.t.)* has not yet been completely demonstrated in relation to deep male genital tract acute and chronic pathologies; the micro-organism has been detected in prostatic secretions, prostatic biopsies, in Sertoli cells from testicular biopsies and in semen. The biology of the micro-organism, the intracellular parasitism and metabolism, influences and determines the infection evolution towards the chronicity and the following tissue inflammation in affected patients. In males the *C.t.* infection has been associated with fertility alterations due to tissue degradation processes, functional or immune related alterations of the genital tract or of the spermatozoa and with pathologies of the upper genital tract such as prostatitis and infertility. *Chlamydia trachomatis* has been additionally reported to act particularly stimulatory for the appearance of the secretary (S)IgA in the genital tract. The intrinsic capacity of the male genital tract to produce SIgA is well documented and can take place both in the prostate and the epididymis, resulting in local targeted overproduction of SIgA by submucosal plasma cells. The induction mechanisms, the regulation of these immunoresponses and the TH immuno-shift in the male genital tract are poorly understood up to now. The overproduction of SIgA could be theoretically explained by release of cytokines such as interferon gamma, interleukin-5 and tumor necrosis factor alpa. Cytokines are important mediators in inflammatory processes and their production may modulate immunity during *C. t.* infections, as proved by recent in vitro studies. In our previous studies we proved elevated IL-6 concentrations and overproduction in semen of chronic prostatitis patients.

**Aim of our study**: We investigated on IL-6, IL-4, IL-10 mucosal presence in relation to *C. t.* specific IgA, SIgA and IgG content in seminal fluids of 110 men affected by prostatitis. Additional objective was to determine the possible TH response and the possible correlation between cytokine production and the presence of *Chlamydia trachomatis* DNA in semen of the patients.

**Study population**: Patients' study inclusion criteria were the presence of acute or chronic prostatitis; the population has been select in a study period of seven months from November 1995 to June 1996. Patients' symptoms were urethrorrea, pollachiuria, stranguria, haemospermia, perineal pains, tenesmus and infertility (8.2%); patients ranged from 21 to 73 years-old (m age=39.5). *C. t.* was searched in patients semen by a modified PCR (Roche D.S., Germany). Specific anti *C. t.* IgA were detected by IP (Savyon D. Ltd., Israel), MIF (Labsystem, Finland) an anti *C. t.* MOMP ELISA test (SeroCT by Savyon D. Ltd., Israel) and IgG were detected by SeroCT, Savyon D. Ltd., Israel. IL-6, IL-4, IL-10 were detected by research quantitative ELISA tests

(Biokine, T-Cell Diagn. Inc. and Predicta Genzyme, USA).

**Results:** Sperm *C.t.* DNA was present in 13/110 patients (11.8%). IgA by MIF and HP were present in 40/110 patients (36.36%). IgA anti MOMP detected by an ELISA test were present in 16/110 patients (14.5%). Sperm *C. t.* MOMP IgG was demonstrated by SeroCT ELISA in none of the patients excluding the possible transmembrane translocation from sera of these antibodies or the local production demonstrating the long-term infections. Western-blot analysis of semen and serum IgA and IgG immunoresponse proved a specific immunization versus *C. t.* specific proteins, both in *C. t.* DNA positive pts. and in *C.t.* IgA positive pts. IL-6 was present at concentration >10 pg/ml in ejaculates of 10/110 (9.1%) patients; IL-10 was present at concentration >10 pg/ml in 23/110 (20.9%). A good correlation was found between IL-6 and IL-10 production (r=0.623) in these patients. IL-4 was not measured at detectable levels in ejaculates of our patients. Thus may be due to its rapid metabolism.

**Conclusions:** Our patients with anti C. t. IgA positive prostatitis have DNA proven "Chlamydial" prostatitis in 30.7%. They presented specific local immunization against the micro-organism western-blot proved, local overproduction of IgA confirming the proper immunocompetency of the genital tract. *C. t.* DNA positivity confirmed the presence of the micro-organism as a possible "primum movens" of the pathology.

Concerning the IL-6 and IL-10 local production it is in vivo well documented in our patients affected by prostatitis, confirming the production of these multifunctional cytokines in human chlamydial prostatic infection, too. 20.5% of the semen IgA positive patients presented high level of IL-10. The presence of IL-10 in ejaculates of our patients seems related to a TH2 shift of the immune response, probably strictly connected with the infection persistence and with possible modifications of the micro-organism (latency?) causing the infection; only one of the semen IgA-IL-10 positive patients demonstrated the presence of plasmidic *C. t.* DNA.

The biological role of IL-6, the presence of other Th2 related cytokines and the secretory IgA overproduction may be important factors in micro-organism clearance, in modulating the passage from acute to chronic prostatic infection and may be involved in the possible determinism of prostate cancer. All these observations seem thus emphasized from our findings confirming the pathogenesis of prostatitis as immuno related.

### Detection Of *C. trachomatis* DNA and Chlamydial Inclusions Using Non Radioactive In Situ Hybridization And Apaap Staining Of Placental Samples

***M Gencay, M Puolakkainen, T Wahlstrom, P Ammala, L Mannonen, A Vaheri, M Koskiniemi***

*Haartman Institute, Department of Virology, and Department of Obstetrics and Gynecology, University of Helsinki, Finland*

**Objectives:** Our aim was to study placental tissue of a stillbirth fetus, born to a mother with high antibody titers to *Chlamydia trachomatis*, using non radioactive RNA:DNA hybridization and alkaline phosphatase-anti-alkaline phosphatase staining (APAAP) for *C. trachomatis.*

**Method:** *In situ hybridization*

For the probe preparation for in situ hybridization, *C. trachomatis* L2 plasmid DNA was subcloned into a polylinker site of a pGEM-3Zf(-) vector [L2PpGEM]. RNA transcripts were synthesized using linearized and purified L2pPGEM as a template. To shorten the probe and to facilitate diffusion into the tissues, probes were hydrolized under alkaline conditions. Nonspecific probe was prepared from vector [l2PpGEM] by the same method as specific probe. Infected and uninfected McCoy cells were used to check performance of the probes. *C. trachomatis* specific and non-specific probes were used for the detection of the *C. trachomatis* DNA in paraffin embedded tissue sections.

*Alkaline phosphatase-Anti-Alkaline phosphatase (APAAP) staining*

APAAP staining of chlamydial inclusions in tissue sections was done as previously described by Mahony (1) with slight modifications and counterstained 10s in Mayer's hematoxylin. To control the nonspecific staining, duplicate tissue sections were incubated with mouse ascites fluid. Slides were examined by light microscope.

**Results:** *C. trachomatis* specific nucleic acid and chlamydial inclusions were detected in placental specimens by in situ hybridization and APAAP staining. Using appropriate negative and positive controls, these tests indicated the presence of *C. trachomatis* in the placenta whereas PCR tests for HSV-1, HSV-1, Varicella-zoster virus and toxoplasma gondii were negative.

**Conclusions:** This is the first case that shows the presence of *C. trachomatis* in human placenta. *C. trachomatis* is the most common sexually transmitted disease and it is usually asymptomatic. *C. trachomatis* in placenta exposes the fetus to the infection and may cause birth complications. These findings stress the need for further studies of *C. trachomatis* infections during pregnancy because specific therapy is available.

**References**

1. Mahony JB, Sellors J, Chernesky MA. Detection of Chlamydial inclusions in cell culture or biopsy tissue by alkaline phosphatase-anti alkalin phosphatase staining. J Clin Microbiol 1987;25:1864-1867.

### Prevalence And Viability Of *Chlamydia trachomatis* In Infertile Women

***HC Gerard, PJ Branigan, SS Minassian, AP Hudson.***

*Allegheny University, Dept. of Microbiology and Immunology and Division of Reproductive Endocrinology and Infertility, Philadelphia, PA 9129*

Previous studies have suggested that the intracellular bacterium *C. trachomatis* is associated with infertility in women. We developed polymerase chain reaction assays targeting the 16S RNA and the major outer membrane protein (MOMP) genes to examine endometrium, fallopian tubes and cervix samples from infertile women. PCR assays use primers targeting the 16S RNA and MOMP genes to amplify chromosomal DNA directly or linked to a reverse transcriptase to analyze 16S RNA primary transcripts and messenger RNA for MOMP. We first used PCR assays to investigate the presence of *C. trachomatis* in subclinical endometrial and tubal chlamydia infection. Seven consecutive endometrial biopsies and ten tubal fallopian samples were obtained for PCR and RT-PCR analysis. Five of seven patients' cervical and endometrial samples were found positive for chlamydial DNA by these PCR assays. Importantly, endometrial samples from two PCR-negative patients (cervix) were PCR positive for chlamydial DNA revealing the presence of *C. trachomatis* in the upper genital tract. We and others reported the presence of DNA from *C. trachomatis* in fallopian tubes from infertile women and we have determined whether *C. trachomatis* is metabolically active during infection. Ten fallopian tubes samples from female patients with ectopic pregnancies were also tested for the presence of DNA from *C. trachomatis* and seven were found positive by both PCR assays. RT-PCR analyses of total RNA from these seven DNA fallopian tubes samples showed 16S RNA primary transcripts and mRNA from MOMP in two of the seven DNA directed PCR positive patients. Control RT-PCRs for host cell actin transcript showed that each RNA preparation was of adequate quality for analysis. These results demonstrated that inapparent chlamydia in fallopian tubes are viable and metabolically active.

---

### Antibodies To Human 60 kDa Heat Shock Protein (hsp60) In Sera Of Women With Pelvic Inflammatory Disease (PID) Correlate With Immune Response to a Conserved B-Cell Epitope Of The *Chlamydia trachomatis* hsp60

***K Bergstrom[1], SS Witkin[2], J Paavonen[3], P-A Mardh[1] and M Domeika[1]***

*[1]Institute of Clinical Bacteriology, Uppsala University, Uppsala, Sweden; [2]Department of Obstetrics and Gynecology, Cornell University Medical College, New York, USA; [3]Helsinki University, Helsinki, Finland*

**Objective:** Antibodies to the *C. trachomatis* hsp60 have been shown in several studies to be associated with PID. It has been hypothesisted that sensitization to the chlamydial hsp60 leads to an immune response to the homologous human hsp60. Tubal occlusion in these patients may be due to an autoimmune response to human hsp60 induced in tubal epithelia. In the present study we examined the association between humoral immunity to two synthetic peptide B-epitopes of the *C. trachomatis* hsp60, one of which is expressed in the human hsp60, and the presence of abs to the human hsp60 in women with PID. Abs in human sera to peptide 260-271, but not abs to peptide 151-162, were previously shown to react with homologous epitopes in human hsp60[1].

**Materials and Methods:** The study population consisted of 129 women with PID. IgG and IgA abs to chlamydial lipopolysaccharide (LPS) were detected as described earlier[2] in modification[3]. IgG abs to synthetic peptides corresponding to amino acids 151-162 and amino acids 260-271 of the chlamydial hsp60, and to purified recombinant human hsp60 were detected by ELISA.

**Results:** IgG abs to human hsp60 were detected in 73 (56.6%) PID patients. The presence of abs to peptide 260-271 was correlated with abs to the human hsp60 ($p=0.011$). Antipeptide 260-271 abs were present in 36 (49.3%) women with, and in 15 (26.8%) women without abs to human hsp60. As expected, abs to synthetic peptide 260-271 were highly associated with presence of IgG ($p=0.0006$) and IgA ($p<0.0001$) abs to the *C. trachomatis* LPS (Table 1). In contrast, there was no association between abs to the chlamydial hsp60 peptide 151-162 which is not expressed in the human hsp60 and immune response to the human hsp60 ($p = 0.018$).

**Conclusions:** Immune sensitization to the human hsp60 was associated with a humoral immune response to a conserved epitope of the chlamydial hsp60 in PID patients with serological evidence of exposure to *C. trachomatis* upper genital tract infection in those women who develop immune response to an epitope of the chlamydial hsp60 cross-reactive to an epitope in the human hsp60.

Table 1. Relation between IgG abs to a conserved *C. trachomatis* hsp60 epitope 260-271, IgG and IgM abs to the lipopolysaccharide (LPS) of *C. trachomatis*, and abs to human hsp60 among 129 women with pelvic inflammatory disease (PID)

| Antibodies assayed | Outcome | No. subjects | No. with abs to peptide 260-271 | p value ($X^2$test) |
|---|---|---|---|---|
| IgG anti-human hsp60 | present | 73 | 36 (49.3%) | 0.011 |
| | absent | 56 | 15 (26.8%) | |
| IgG anti-*C. trachomatis* | present | 98 | 47 (48%) | 0.0006 |
| LPS | absent | 31 | 4 (12.9%) | |
| IgA anti-*C. trachomatis* | present | 78 | 47 (60%) | >0.0001 |
| LPS | absent | 51 | 11 (21.6%) | |

**Referemces:**

1. YI Y, Zhong G, Brunham RC, Continuous B-cell epitopes in *Chlamydia trachomatis* heat shock protein 60. Infect. Immun 1993;61:1117-1120.
2. Brade L, Brunnemann H, Ernst M, et al. Occurrence of antibodies against chlamydial lipopolysaccharide in human sera as measured by ELISA using an artificial glycoconjugate antigen. Fems Immunol Med Microbiol 1994;8(1):27-41.
3. Bergstrom K, Brade L, Witkin SS, Mardh P-A, Brade H, Domeika M. Characterisation of antibody response to chlamydia in patients with sexually acquired reactive arthritis. 1996;(In manuscript).

### Humoral Immunological Response To A Sequence Of The 57kD Heat Shock Protein Common To Mouse And *Chlamydia trachomatis*, in Mice Inoculated With a *Chlamydia trachomatis* E Strain

**MJ Borrego*, V Fuentes, E Bissac, MA Catry, F Eb, J Orfila**
**Laboratorio de Bacteriologia, Instituto Nacional de Saude Dr Ricardo Jorge, Av Padre Cruz 1699 Lisboa codex, Portugal*

*Chlamydia (C). trachomatis* is responsible for human genital tract and eye infections in adults, and for respiratory tract infections in newborn children. Infection of the female genital tract causes cervicitis and salpingitis. The salpingitis due to *C. trachomatis* is considered responsible for ectopic pregnancy and infertility. The pathogenic events that lead to development of these pathologies are not known; as repeated infection seems to play a role, immunologically mediated mechanisms have been suggested. A pathogenic role of delayed hypersensitivity of *Chlamydia* 57kD heat shock protein (HSP) has been proposed in establishing a chronic inflammation, leading to scarring associated with fallopian tube obstruction and subsequent infertility. Various animal models have been used in order to study this phenomenon. Tuffrey's work (Br. J. Exp. Path., 1986) indicated that mouse constitute a good model to study genital infection due to *C trachomatis* human strains. In our study, we used C3H/He and C57/B16 female mice. These strains don't belong to the same H-2 haplotype. Mice were treated with progesterone (1.25mg per mouse) 7 days before intracervical (by vaginal route) inoculation with a *C. trachomatis* genotype E stain isolated from a portuguese patient ($10^4$IFU per mouse). Mice were reinoculated at days 39, 68, 94 131 and 164 after the first inoculation. At days 5, 18, 22, 29 and 36 after inoculation and 1 month after each reinoculation, a blood sample was taken from 5 mice of each strain before sacrifice and research for *C. trachomatis* in the different sections of the genital tract by culture and PCR-AMPLICOR-COBAS^ROCHETM. Mice were also studied for fertility at days 22, 36, 65, 92, 127, 162 and 191 of the experimental procedure. Control animals were inoculated and reinoculated with a L929 cell supernatant Blood samples, *C. trachomatis* research and fertility studies were done as for inoculated mice. A peptide (E-10-A) chosen from a common sequence of *Chlamydia* and mouse 57kD HSP by epitope plates (25µl/ml) and reactivity of mouse IgG antibody was analysed We also observed the sera reaction to a recombinant 57kD HSP by Western-blot. The microganism was detected by culture in the ovarian and fallopian tubes at day 5 and the AMPLICOR-COBAS^ROACHETM could detect the agent up to day 190 of experiment. A serological reaction to the E-10-A peptide and to the *Chlamydia* recombinant 57kD HSP could be observed since day 5 in inoculated animals, and particularly in the C3H/He mice. However no relation between this humoral immunological response and fertility could be established.

**References**
Br. J. Exp. Path., 1986, 67, 605-616.
J. Inf. Dis., 1993, 168, 1236-1240.
J. Inf. Dis., 1994, 169, 680-683.
Trends in Microbiology, 1994, 2, 94-98.
J. Inf. Dis., 1987, 155, 1292-1299.
Inf. Imm., 1992, 60, 3143-3149.
J. Exp. Med., 1989, 170, 1271-1283.

### The Presence Of Serum Antibody To The Chlamydial Heat Shock Protein (CHSP60)

**[1]P Claman, [1]L Honey, [2]R Peeling, [3]P Jessamine [4]B Toye**
*[1]Division of Reproductive Endocrinology, Dept. of Obstetrics & Gynecology, Ottawa Civic Hospital, University of Ottawa, [2]Laboratory Centre for Disease Control, Winnipeg Manitoba, [3]Division of Microbiology and immunology, Department of Medicine, Ottawa Civic Hosspital, University of Ottawa, [4]Division of Microbiology, Department of Pathology and Laboratory Medicine, Division of Infectious Disease, Department of Medicine, Ottawa General Hospital, University of Ottawa.*

**Objective**: To study the utility of testing for *Chlamydia* heat shock protein 60 (CHSP60) antibodies in the diagnosis of tubal factor infertility (TFI).

**Design**: Prospective case control.

**Setting**: Canadian University Hospital Infertility Clinic

**Main outcome measures**: Sera was collected from 77 women presenting for infertility investigation and analyzed for antibodies to both *C. trachomatis* and CHSP60.

**Result**: Antibodies to *C. trachomatis* by microimmunofluorescence (MIF) were found in 10 of 16 (63%) women with TFI but also in 28 of 61 (46%) women with other causes of infertility (p=0.2, OR=1.96, 95%CI=0.56-7.1). Seven of 16 (44%) women with TFI and only 5 of 61 (8%) women with other causes of infertility had anti CHSP60 antibodies (p=0.002, OR=8.7, 95% CI=1.9-41.9). MIF testing has a 63% sensitivity and a 54% specificity for detecting the presence of TFI. A patient with TFI is only 1.36 times more likely to have a positive MIF test than a patient without TFI (positive Likelihood Ratio). In contrast, the CHSP60 antibody test has a 44% sensitivity and a 92% specificity for the presence of TFI. A patient with TFI is 5.5 times more likely than a patient without TFI to have antibodies to CHSP60 (positive Likelihood Ratio). MIF and CHSP60 testing in combination has a positive likelihood ratio of 10 for the detection of *C. trachomatis* associated TFI.

**Conclusions**: Antibody testing by MIF for *C. trachomatis* alone is a poor test for predicting the diagnosis of TFI. CHSP60 antibody testing is a more accurate test than MIF in predicting *Chlamydia* associated TFI. These tests should be used in combination as part of an initial infertility evaulation to provide a rapid means of diagnosis and to save on unnecessary and costly interventive procedures.

### Functional Damage of the Fallopian Tubes and Immune Responses To *Chlamydia trachomatis*

***I Sziller*, SS Witkin**, M Ziegert**, P Fedorcsak*, Z Papp*.***

**Department of Obstetrics and Gynecology, Semmelweis University Medical School, Budapest, Hungary and **Division of Immunology and Infectious Diseases, Department of Obstetrics and Gynecology, Cornell University Medical College, New York, USA*

**Objectives:**To determine serologic responses of patients with impaired transport functions of the Fallopian tubes to two Chlamydial antigens - L-2 and 60 kDa heat shock protein - in a Hungarian University setting.

**Design:** In a case-control study, serologic responses to chlamydial antigens were examined in five groups of patients. Group 1 consisted of 28 patients with ectopic pregnancy and histologically proven chronic salpingitis, while Group II included 40 patients treated for ectopic pregnancy without histologic signs of prior tubal infection. In Group III, 41 patients seeking care for in-vitro fertilization were enrolled with laparoscopically proven tubal factor infertility. Two groups of patients served as controls: Group IV consisted of 12 infertile patients in whom impaired tubal transport function was due to pelvic endometriosis as evidenced by laparoscopy, and Group V included 45 patients with an uncomplicated mid-trimester pregnancy. In sera of patients seropositive for *Chlamydia trachomatis*, serologic responses to the 60 kDa chlamydial heat shock protein were also determined.

**Results:** The prevalence of anti-chlamydial IgG among patients with salpingitis-associated ectopic pregnancy, in women with ectopic pregnancy without histologic evidence of chronic salpingitis, and among those with infection-related tubal infertility was 68% (19/28), 38% (15/40), and 44% (18/41), respectively. In the control groups 3 (25%) of 12 patients with pelvic endometriosis leading to impaired tubal patency and 17 (27%) of 64 pregnant had anti-chlamydial IgG detected. Sera of patients seropositive for *Chlamydia trachomatis* were also evaluated for the presence of antibodies to the 60kDa chlamydial heat shock protein. The prevalence of ajti-HSP antibodies was 63% (12/19) in Group I, 40% to the 60kDa chlamydial heat shock protein. The prevalence of anti-HSP antibodies (6/15) in Group II, 39% (7/18) in Group III, o% (0/3) in Group IV, and 6% (1/17) in Group V, respectively.

**Conclusions:** Besides a significantly higher prevalence of anti-chlamydial antibodies to a genus chlamydial antigen, patients with tubal damage leading to ectopic gestation or tubal infertility were more likely to have antibodies to the 60 kDa chlamydial heat shock protein than those with an uncomplicated pregnancy or women with impaired tubal transport due to pelvic endometriosis. Our data suggest that prior chlamydial infection is associated with an increased risk of tubal infertility and ectopic pregnancy also in Hungary. In addition, the presence of antibodies to the chlamydial heat shock protein is a marker of infection-related tubal damage.

### Production Of IL1-β In Response to Chlamydial Infection in an In Vitro Model of The Human Fallopian Tube

***KA Ault, O Tawfik, MM Smith King and J Gunter***

*University of Iowa Hospitals and Clinics, Iowa City, IA and University of Kansas Medical Center Kansas City, KS.*

**Objectives:** Cytokines are known to be important in both the inflammatory response and antigen expression in chlamydial infections. Interleukin 1 beta (IL1-β) is an especially important cytokine because of its multiple pro-inflammatory and fibrogenic properties. It is our hypothesis that IL1-β will be produced in response to chlamydial infection in an *in vitro* model of the human fallopian tube.

**Methods:** Tubal segments are harvested at the time of abdominal hysterectomy and processed using standard tissue culture techniques. Three 4mm biopsies were taken from the lumen of the fallopian tube. Two of these were inoculated with 50 microliters of a $1 \times 10^8$ solution of *Chlamydia trachomatis* serotype E/UW-5CX elementary bodies and one was "sham" inoculated. After forty-eight hours, supernatant was assayed for IL1-β using an enzyme linked immunoabsorbent assay (Cytoscreen Immunoassay Kit, Biosource International Camarillo CA). Additionally, tubal segments were stained for IL1-β using immunohistochemical techniques with a polyclonal rabbit anti-human IL1-B. (Genzyme, Cambridge MA).

**Results:** Seven tubal segments were cultured. Mean IL1-B levels for infected segments were significantly higher at 69.54 pg/ml +/- 41.0 (mean +/- standard deviation) versus control values of 2.4 pg/ml +/- 0.46 ($p<0.005$ with paired t test). IL1-β was predominantly localized in the tubal epithelium. Other areas with IL1-β included stromal cells morphologically consistent with macrophages and fibroblasts.

**Conclusions:** The human fallopian tube produces IL1-β in response to infection with *Chlamydia trachomatis.* Because of its biological properties, IL1-β may play a significant role in the early inflammatory response of the human fallopian tube as well as the eventual pathogenesis of tubal damage.

### Interest of Detection Of Antibodies In Ct Positive And Ct Negative Patients With Genital Infection

***J Orfila, C Chaigneau, JM Sueur***

*Biobanque de Picardie - Amiens, France*

**Patients**: 59 women and 19 men consulting for STD detection. The clinical symptom varying from acute lower genital infection to acute and chronic higher genital infection.

**Protocol**: All patients were submitted to urethral or cervical collections in the following order:

- first swab for cell culture on McCoy cells
- second swab for LCR (Abbott lab)
- third swab for direct immunofluorescence (DFA) with monoclonal antibodies (Syva)
- fourth swab for detection of secretory IgA (Medac)

Blood sample were taken the same day in order to detect IgG, IgM, IgA. Three different techniques have been used: microimmunofluorescence with three antigens (*C. trachomatis, C. psittaci, C. pneumoniae* ELISA (Medac) using a recombinant LPS, ELISA (Orgenix) with two antigens (*C. trachomatis, C. pneumoniae*) treated in order to eliminate the LPS.

**Results**: *C. trachomatis* has been found in 22 patients (group 1), 56 were negative by all technics (group II). Secretory IgA were found for 11 patients from group I (50%) and 6 from group II (10.7%) with a significant difference between the two groups p=0.00005.

Table I. The result obtained with the three techniques for serum antibodies.

| | | Group I | Group II |
|---|---|---|---|
| IgG | Medac | 86.36 (19/22) | 82.14 (46/56) |
| | M.I.F. | 81.81 (18/22) | 73.21 (41/56) |
| | PBS | 72.72 (16/22) | 51.78 (29/56) |
| IgM | Medac | 13.63 (3/22) | 26.78 (15/56) |
| | M.I.F. | 27.70 (6/22) | 10.71 (6/56) |
| IgA | Medac | 40.90 (9/22) | 48.10 (27/56) |
| | M.I.F. | 18.18 (4/22) | 16.07 (9/56) |
| IgA | Secretory | 50 (11/22) | 10.71 (6/56) |

The analysis of the different results shows that MIF and Medac ELISA have similar sensitivity with two patients with ELISA + and MIF - at the first visit. The two tests becoming positive at the second visit. On the other hand with Orgenix 2 patients positive with MIF and ELISA are negative at the first visit. They became + with the three tests later. It seems that anti LPS antibodies are the first to appear or at least the first to be detected. However, the small number of patients ask for a deeper investigation. Considering the IgA humoral response we may describe two types of response: 1) In case of acute infection: 3 IgA sero conversions were noted a fortnight after contamination and after IgG sero conversion. After treatment IgA sero negotiation has been noticed. 2) In case of chronic infection: the presence of IgA was always linked to persistent upper chronic infection. Secretory IgA: In spite of technical difficulties (the use of the fourth sample) the results are rather satisfactory. S IgA was present in 50% of positive direct im examination. With treatment secretory IgA become negative in a few weeks.

### Clinical Research Of *Chlamydia Trachomatis* Infection In Women's Genitals

***XS Wen, YJ Lin, G Zhu***

*Dept. of Ob/Gyn, The First Affiliated Hospital, Harbin Medical University, P.R. China*

From July 1993 to July 1995, we detected CT in 1487 gynecological outpatients by PCR (Polymerse Chain Reaction). We also detected the response to therapy and the host's immunological response among the 218 patients who were positive for CT. The total positive rate was 26.09% (388/1487). The risk age was from 21 to 35. We divided 218 patients into 3 groups and used 3 different treatments, which were antibiotics, Chinese drugs and antibiotic combined with Chinese drugs. The cure rate was 75% (45/60), 65.86 (48/75) and 92.86% (78/84) respectively. We compared antibiotic therapy with Chinese Western drugs therapy by $x^2$ test, $x^2$=8.69, $p < 0.01$, the comparison between Chinese drugs and Chinese Western drugs showed x2 =19.08, p<0.01. The results demonstrated the difference among these three methods was obvious, the best was Chinese - Western drugs therapy. We also compared the difference between before - therapy level of IgA, IgG and after - therapy level of IgA, IgG by t test, $t_A$=5.228, $t_G$=3.175, p<0.05, the difference was also obvious. This result response which was not specific according to the variety of antibody, so the level of antibody was only regarded as a reference therapeutic evaluation parameter.

### Atypical Morphology of *Chlamydia trachomatis* in Patients With Latent Infection

***EE Bragina, MA Gomberg, OE Orlova***

*Moscow, Russia*

The aim of this study was to evaluate in vivo morphologic changes of *Chlamydia trachomatis* in order to explain the ineffectiveness of antibiotic treatment in some patients with chronic *chlamydia* infection. At study entry chlamydial infection was diagnosed by direct immunofluorescence (IF) and by cell culture (line L929) with IF confirmation. Small atypical cytoplasmic inclusions (SACI) containing chlamydial antigen were isolated in some cases. The cultured cells usually contained several SACI around the cell nucleus, and they did not enlarge during cultivation. They correlated with inclusions described earlier for an in vitro model of persistent chlamydial infection. In our study SACI were isolated from 16 patients (9 men and 7 women) 7-10 days after unsuccessful treatment with routine methods. Chlamydia in SACI continued to be isolated 6 weeks after treatment in 10 out of 16 patients and disappeared spontaneously in 2. One month later inclusions disappeared in another 3 patients, but appeared again in one. We performed an ultrastructure study of material from these patients. Different stages of chlamydial life cycle were revealed: adhesion of elementary bodies (EB), intracellular inclusions, the export of intracellular *C. trachomatis* out of the host-cells. Only 2 small cytoplasmic inclusions were found and both contained exclusively reticulate bodies (RB) without any matured Ebs. Some of the RBs were at the stage of division and in some others condensation centers were observed. We considered these inclusions as SACI revealed in culture cells after isolation from patients in whom infection persisted after unsuccessful antibiotic therapy. In latency that clinically could not be distinguished from persistency, chlamydia do not demonstrate any metabolic processes. The cases of chlamydial latency are illustrated with extracellular mono- and polymembrane vacuoles with chlamydial Ebs inside, or one EB, surrounded with additional membrane layers. In some cases we have demonstrated that these additional outer membranes are originating from the host-cell membranes. These membranes may prevent adhesion of EBs to the host-cell and determine one of the ways of development of latency in the early stages of the developmental cycle. Recently, some data appeared concerning extracellular division of chlamydial RBs in vitro. We have found host-free dividing Rbs in vivo that were morphologically different from the typical intracellular ones. The outer membranes of the RBs with several atypical condensation centres are separated from the inner membranes and the protoplasm is dividing inside enhanced periplasma spaces. Findings of morphological changes of chlamydial bodies and their interactions with the host-cells may explain the cases of persistence after antibiotic treatment.

### Immunologic Changes In Patients With Persistent Chlamydial Infection

**MA Gomberg, AM Solovyov**
*Department of Dermatology and Venerology Semashko Moscow Medical and Dental School, Moscow, Russia*

It was shown earlier in vitro that some factors of immune system may induce a persistence of *Chlamydia trachomatis*. We estimated different immunologic data in 54 patients with chronic persistent urogenital chlamydial infection. Persistence was confirmed by the atypical small forms of chlamydial inclusions consisting of non-developing reticulate bodies in a cell-culture test (line L-929). According to the results of the study of antigenic landscape of the lymphocytes and the level of immunoglobulins in the peripheral blood all patients were divided into 2 groups. Group 1 consisted of 39 patients with decreased index in their immune status. Group 2 was formed with 15 patients in whom the results of the immunologic study were normal or even higher. In Group 1 immunostimulative therapy was used in 26 patients and the remaining 13 did not receive the kind of treatment as well as all the 15 patients from Group 2. Nobody with abnormal forms of inclusions received antibiotic treatment. In comparison with healthy volunteers there were found various changes in T-cell immunity and significantly decreased levels of CD72+, CD21+, CD16+ and HLA DR+ in patients with persistent chlamydial infection. Most patient of Group 2 showed significantly increased levels of CD3+, CD4+, IR1, CD72+ and IgM. Culture tests were repeated 1-2 months after persistent forms of *C. trachomatis* had first been revealed. Seven out of 13 patients from Group 1 who had not received immunostimulative treatment had no *C. trachomatis* in the second test, and in 6 *C. trachomatis* still persisted. Eighteen out of 26 cases from the Group 1, who had received immunotherapy, *C. trachomatis* disappeared, in 3 cases they transformed into common forms, and in the remaining 5 Chlamydia still persisted. Twelve out of 15 cases from the Group 2 *C. trachomatis* eliminated, 1 patient showed a transformation of chlamydia into common forms and in 2 cases they persisted. So, *C. trachomatis* disappeared in 77% of cases from Group 1 after immunotherapy, in 54% of cases from that group when therapy had not been applied and in 87% of cases when from Group 2 were immunostimulative therapy was not necessary. The disturbances in immune system may cause non-complete climination of the persistent microorganisms. Disappearance of persistent forms may be spontaneous.

### The Immune Response In Chronic Chlamydious And Chlamydious-Herpetic Infection of the Uro-Genital System

**V Danilejchenko, N Vynograd, V Chopjak**
*Department of Microbiology, Lviv Medical Institute, Ukraine*

The indices of cell and humoral immunity as well as interferon status of 44 patients with chronic uro-genital chlamydiasis and 35 patients affected with chlamydious herpetic infection were studied. The analysis of data in both groups of patients shows the presence of secondary cell-stipulated state of immune deficiency which was more marked in case of mixed infection. The course of the illness was followed by a decrease of T-cells, an increase in the activity of T-h and T-s subpopulations of lymphocytes. The increase of relative and absolute count of O-lymphocytes was observed. The changes in humoral link of immunity were characterized by increases in the level of circulating immune complexes and IgG. The active expenditure of complement has been observed on both groups. This can be proved by the fall of complementary activity of blood serum. The study of interferon status of both groups, patients showed more considerable depression of interferon producing activity of leukocytes in patients with mixed chlamydious-herpetic infection. All these patients had much lower ability to produce IFN$\alpha$ and IFN$\gamma$. The disturbances in the immune and interferon systems in patients with chlamydiasis and herpetic chlamydious infections mark their significance in the pathogenesis of these infections the necessity of further researches of new effective methods of treatment.

### Peculiarities Of The State Of The Immune System In Girls With Inflammatory Diseases Of Genitals Of Chlamydial Etiology

***LA Matitsina***

*Donetsk Regional Centre of Maternity and Childhood Protection, Donetsk, Ukraine*

Urogenital chlamydiosis is at present a serious pathology not only in women but also in girls. The pathogen of the given disease - *Chlamydia trachomatis* has tropism to cylindrical epithelium covering not only cervical channel and urethra tubes. We studied a group of girls of 86 persons aged from 7 to 18 with determined diagnosis - urogenital chlamydiosis. This group consisted of girls who were not treated at the moment of study. All girls with chlamydiosis were diagnosed as monoinfection after taking a tage of urethra with subsequent macroscanning by method of indirect fluorescence (with use of test-system "Chlamiscanu") and coloring of the material according to Romanoviski-Gemze. 69 (80%) patients were troubled by genital discharge during 3-5 months. Discharge of white or yellow color was not profuse. 52 (60%) patients were troubled by periodic pains at low art of abdomen during 2-5 months. Rectal examination of adnexia was determined, painfulness, increase of their sizes. Ultrasonic examination availability of inflammatory process of, internal genitals (adnexitis, hydrosalpinz one or bilateral) in 73 (65.8%) girls found that was displayed by the increase of ovary, the decrease of echogenity, the expansion of uterine tubes due to liguid component. Hydrosalpinx is found out in 22 (30.9%) patients, adnexitis in 24 (32.9%) salpingooforitis in 6 (7%) patients, cystis - 4 (5.5%), endometritis 5 (6.9%). Thus, urogenital chlamydiosis is a reason for frequent complications on the part of internal genitals firstly uterine tubes, taking into account the trobity of pathogen to their cylindric epithelium,. Prolonged inflammation in uterine tubes can result in narrowing of their lumen, total obliteration and occurrence herein after tubal sterility. In present time inflammatory process of internal genitals of chlamydial ethnology take a special place. According to the modern view chronic process in internal organs direct pathological influence for all system of organism and lead to disorder of immunological parameters. This immunological disorders of character of disease, time of its course and also influence on the diagnostics and treatment. We studied immunological status in 26 patients (girls of 7-13 years) with chronical disease of internal genitales (chronical adnexitis, chronical salpyngitis) and chronical vulvovaginitis. Immunological status in these patients was escalated before the beginning of treatment. It was determined that on this stage the level of T-lymphocytes averages 68.6 + 3.4% (range 69-85%) level B-lymphocytes 12.6 + 0.9% (in normal 110-17%), phagocytic activity of neutrophils nitrofils (NST - test for B.H. Park, 1968) average 1.4 + 0.1% (in normal 11.20 - 1.52; 48.0 - 54.0%), the level IgA consist in average 1.86 + 0.2g/l (in normal 11.0 -2.6g/1); IgG averages 12.07 + 1.96 (in normal 8.0 + 14.3); level IgM - 2.18 + 0.71, in normal 0.8 -1.4); the level IgE averages 258.6 + 5.6 ng/ml (30 - 350 mmg/ml), CIC averages 81.6 + 2.9 (in normal 40-70) In all patient we investigate the level of secretary IgA in vaginal secrete and this level averages 0.27 + 0.03 (was increased). In all patients the level of lymphocytes in peripheral blood was increased too and consisted 44.2 + 4.6. The content of IgM in patients with urogenital infections was increased. In patients with chronic imflammatory diseases of genitals, produced by CT, the absence of changes in the condition of the cellular link of immunity may be connected with low immunogenity of CT and peculiarities of the vital cycle of the agent. It should be noted the increase of IgM in numerous cases, that confirm the activation of B-lymphocytes by chlamydia. Our findings are coodinated with the results, received by other authors earlier who established that CT in the system in vitro indicated proliferation of B-lymphocytes of peripheral blood and in the presence of T-lymphocytes differentiation of B-lymphocytes into cells secreting immunoglobulins. Thus, in girls with inflammatory disease in internal genitals the increase of the IgM levels in serum and levels of circulating immune complex (CIC) and others immunological parameters are marked. The received data have allowed us to conclude about the efficiency of application of immunological methods of investigation to inflammatory disease of internal genitals.

### Diagnostics And Treatment Of Chlamydia Infection In Women With Preterm Labor

***TN Dyomina, IB Yakovleva***

*Donetsk State Medical University, Donetsk, Ukraine*

From 0.5 to 39% of women with genital inflammation also have chlamydia infection, but one's role in the habitual preterm labor (HPL) is not well known. The aim of this paper was to determine the share of women with chlamydia during HPL. The informativity of the hybridization revealing chlamydial DNA and RCC methods and connection between endometrial and placenta contamination were also studied. We examined 87 patients with HPL (from 2 to 10 abortions) ages 18 to 36. Chronic genital inflammation was revealed in 74 women. Chorion and placenta were analyzed after abortus in 8-24 weeks in 12 patients. The further examination of samples were made after 2-4 months in the first phase of the menstrual cycle. Samples were taken from the cervical canal and urethra by Folkman's spoon. RCC is based upon calculation of the amount of complement which is consumed by the antigen-antibody complex. The antigen is the ornithosic antigen. In the method of the hybridization revealing chlamydia DNA, the technique of pointal hybridization of nucleic acids on solid phase with

DNA-Zonde marked with biotin was used.

The analysis includes several steps:

1. Preparation of the nitrocellulose filter, which is used as a solid phase, to hybridization. The filter was moistened in distilled water, then in NaCl and HNO3 solutions, then one was dried in the air.
2. The preparation of the samples. The suspension of cells of the analyzing material was mixed with the lysing solution, then run 2 hours at 55° C. After centrifugation, the over-fallout liquid was taken into a clean test tube, then ethyl alcohol was added. It ran one night at minus 20° C. After this, the sample was centrifuged. Over-fallout liquid was poured off, the solid fallout was dried in air and dissolved in a small amount of the salt solution. The samples and the control samples were denatured in boiling water bath, then the test tubes were quickly removed into the ice water bath and deposited onto the filter. The filter was dried in the air and baked in 80° C during 2 hours.
3. Hybridization. The filter with samples was soaked in distilled water, put into the petri dish and covered with prehybridization solution. The filter was incubated during 2 hours. Then the prehybridization solution was poured out, and the hybridization solution was added. Hybridization ran during 1 night. Then the filter was cleaned out from the unreacted zonde. The filter was plunged into blocking solution to prevent nonspecific conjugate binding, then it was put into conjugate solution. After this, the filter was cleaned one more time with a special solution, then it was covered with revealing solution and run until there was colour in the spot with the positive control. The filter was cleaned with distilled water and dried.
4. Results. The hybridization results were evaluated visually by comparison with the colour of the control samples. In proper reaction technique, the positive control spot is blue. The negative control spot is colourless. Otherwise the test must be made fresh. The intensive coloured samples are treated as positive samples.

To diagnose chlamydia infected abortuses, 1 x 1 x 1 cm pieces of placenta or chorial tissue were taken. Then the tissue was dissected through all layers and from the dissection surface the tissue samples were taken to analyze chlamydia by revealing DNA of the exciter.

During analysis of the cervical canal and urethra, we found that 27 (37%) of women were infected in RCC reaction and 38 (43.7%) in the DNA hybridization reaction. During localization, 14 patients had injury of the cervical canal or urethra, and 18 had injury of the cervical canal only. The women with chronic relapsing inflammation of uterine appendages had chlamydia infection more often than other women in the analyzing group. Chlamydia was bound with chorion and placenta in 4 samples from 12. The DNA revealing method is more effective than RCC. The DNA revealing method reveals chlamydia in 95.4 %. All women with chlamydia and their sexual partners were thoroughly treated. Patients received drugs which had influence over immune reactivity (tactivin and timolin), biostimulators (aloe, plasmol, hyaloid body), pirogenal or prodigiosan, vitamins R, C, methyluracil. If the temperature reaction had occurred, patients received the tetracycline series antibiotics, eritromycin, cyprobai, sumamed with trichopol (after 8 days of therapy). Patients also received antomycotic drugs - nistatin, levorin, clotrimasol. From the 3rd day, the vagina had been sanated by anticeptical solutions (protargol, furacillin) and complex gels with tetracyclin and dimexid. During the 3rd week, patients received physiotherapeutic methods (ultrasound to the bottom of stomach, dyodinamic current, low-intensive laser beams to vagina and the uterus neck, variable zone decompression). After therapy, chlamydia was revealed in 4 women, which received the therapy one more time. 18 women after therapy were pregnant and had normal labour and healthy babies. So, 32.4% of women with chronic relapsing inflammation of uterine appendages also had chlamydia infection.

---

### *Chlamydia trachomatis* Cervical Infections: Some Characteristics

***IM Linhares, SD Miranda, AM Fonseca, EG Percyra, VR Bagnoli, RG Almeida, H Melles, LFG Siqueira***

*STD/AIDS Service, Gynecol/Obstet. Dept., Sao Paulo University Medical School, Brazil*

**Objective:** The aim of this study was to contribute to the knowledge of the prevalence, epidemiological characteristics and clinical characteristics of *Chlamydia trachomatis* cervical infections.

**Methodology:** We studied 166 women complaining of abnormal vaginal discharge (group A) and 25 women without complaint of vaginal discharge (group B). All patients were submitted to culture for *Chlamydia trachomatis* in cervical secretion. For the women whose cultures were positive we analyzed age, race, complaint of pelvic pain, pain at sexual intercourse, sexual antecedents, gynecological examinations characteristics, vaginal pH and Gram stain.

**Results:** The *Chlamydia trachomatis* culture was positive in 15(9.0%) patients of group A and in 3(12.0%) of group B. For these positive cases, the age varied from 14 to 51 years (x=33.7) being 12 patients white and 3 black in the group A. In group B the age varied from 22 to 41 years (x=28.6) and all the patients were white. Pelvic pain and pain at sexual intercourse were referred respectively for 8(53.4%) and 7(46.7%) patients of group A and for 1(33.3%) and 1(33.3%) patient of group B. The sexual antecedents, gynecological examination characteristics, vaginal pH and Gram stain of the both groups are representing in the tables 1, 2, 3 and 4, respectively.

Table 1 - Sexual Antecedents

| | Group A | Group B |
|---|---|---|
| First intercourse | 12-29 years(x=18.3) | 16-33 years(x=22.3) |
| Sexual habits | 100% heterosexual | 100% heterosexual |
| One sexual partner | 10(66.6%) | 3(100%) |
| Two sexual partners | 3(20.0%) | 3(66.6%) |
| Three sexual partner | 2(13.4%) | —— |

Table 2 - Gynecological examination characteristics

| | Group A | Group B |
|---|---|---|
| Painful cervical mobilization | 6(40.0%) | — |
| Presence of vaginal discharge | 5(33.3%) | 1(33.4%) |
| Painful anexial examination | 4(26.6%) | — |
| Presence of cervical discharge | 1(6.6%) | — |

Table 3 -

| Vaginal pH | Group A | Group B |
|---|---|---|
| 4.0 | — | 1(33.3%) |
| 4.5 | 6(46.1%) | 2(66.6%) |
| 5.0 | 1(7.7%) | — |
| 5.5 | 4(30.8%) | — |
| 6.0 | 1(7.7%) | — |
| 6.5 | 1(7.7%) | — |

Table 4 - Vaginal Gram stain

| | Group A | | | | Group B | | | |
|---|---|---|---|---|---|---|---|---|
| | A | R | S | N | A | R | S | N |
| leucocytes | 1 | 6 | 6 | 2 | - | 1 | 2 | - |
| Dodericin bacilli | 6 | - | 2 | 7 | - | - | 1 | 2 |
| Gram positive cocci | - | 9 | 6 | - | - | 3 | - | - |
| Gram negative bacilli | 5 | 8 | 2 | - | 2 | 1 | - | - |

A= Absence; R= Rare; S = Some; N = Numerous.

**Conclusions:**

1. The prevalence of *Chlamydia trachomatis* was similar in symptomatic and asymptomatic women.
2. The cervical infection was observed in young women and women over 40 years old.
3. The absence of clinical complaints do not exclude the possibility of *Chlamydia trachomatis* presence.
4. The same considerations are valid for women who have only one sexual partner.
5. The gynecological examination for the patients with cervical *Chlamydia trachomatis* may present different characteristics or not show alterations.
6. The Gram stain does not offer any indication of the infection.
7. Considering the serious consequences of the complications for women's health of a silent infection, the authors suggest the necessity of periodical screening.

### The Investigation Of Chlamydial Infection In Tubal Infertility

***JA Tait, J Hewitt, W Tong***

*Liverpool Women's Hospital, Liverpool, United Kingdom*

**Objective:** To find, using PCR gene amplification technique, what proportion of infertile women with tubal obstruction, (excluding endometriosis), have evidence of *Chlamydia trachomatis* (Ct) infection in biopsy material from the tubes. Pervious work, using different tests, revealed evidence of persistent chlamydial infection in similar women with a history of treatment.

**Subjects:** Ten infertile women, with or without a past history of pelvic inflammatory disease, lower genital tract chlamydial infection or episodes of pelvic pain, without clinical signs of upper or lower genital tract infection, found on previous laparoscopy to have tubal disease, undergoing laparoscopy to assess and repair the tubal damage.

**Method:** Immediately prior to laparoscopy, a routine endocervical chlamydial swab and serological test for antichlamydial antibodies were taken. Minimal tissue biopsies were taken from the membrane and adhesions bilaterally and tested for Ct by modified PCR. (As these modifications are untested, we cannot use them as a basis for treatment and so all patients will be offered postoperative azithromycin, as wEll as their partners). Delay in obtaining Ethical Committee approval, now secured, first patients to be seen this week.

**Comment:** If the anticipated persistent cases of chlamydial infection are found, preoperative effective antichlamydial treatment will be indicated for all such cases to prevent reactivation after surgery.

### Problems of Diagnosis and Treatment of Genital Chlamydia Infections In Latvia

***[1]G Lazdane, [2]P-A Mardh***

*[1]Department of Ob/Gyn, Medical Academy of Latvia,*
*[2]Institute of Clinical Bacteriology, Uppsala University, Sweden*

**Objective:** To analyze the incidence and the cases of misdiagnosis of genital chlamydia infections in Latvia as well as the methods of treatment used. The work was performed as a part of the evaluation of the situation of the reproductive health of women and the medical care assessment in Latvia.

**Methods:** We analyzed data from State Centre for Sexually Transmitted Diseases of Latvia and Department of Social Statistics of Ministry of Welfare of Latvia. We compared the data mentioned in the latest medical publications in Latvia (1993-1996) dealing with the problems of genital chlamydiosis with the results obtained from the collaboration study with the Institute of Clinical Bacteriology of Uppsala University "The epidemiology of genital Infections in Europe". This study included PCR diagnostics of urine samples of 200 women attending the family planning centre. The retrospective analysis of the case histories of 100 cases of female genital chlamydial infection attending 7 different out-patient clinics in Riga was performed using a special questionnaire.

**Results:** Genital chlamydiosis is one of the reportable sexually transmitted diseases in Latvia and its incidence rate is growing (1992 - 832; 1995 - 4520 cases). The existing medical care system where the costs of laboratory tests are not covered by the sick fund influences the quality of diagnosis and appropriate treatment. There is a great difference in the incidence rate of Chlamydia positive patients reported by the laboratories performing the tests (up to 60-70% Chlamydia positive clients) with the data obtained during the collaborative study with the Uppsala University (3.8% - Chlamydia positive women). When analysing the case histories of the treated patients there were errors of diagnostic, management and treatment in 38% of cases.

**Conclusions:**

1. Due to the use of unspecific methods of diagnosis of genital chlamydial infections in Latvia, the statistical data characterizing this effect in Latvia are not reliable.
2. The cases of false-positive results and unnecessary treatment happen in more than one third of the reported cases of genital chlamydiosis in Latvia.
3. The government of Latvia is to review the state budget for health care and provide means to cover the expenses of specific methods of diagnostic of *Chlamydia trachomatis* infection.
4. Specialized training in detection and counseling on genital chlamydial infections should be organized for health care providers in Latvia that would include assessment of obtained knowledge.

---

### Detection of IgM and IgG Antichlamydial Antibodies in Patients With Nongonococcal Urethritis Caused by *Chlamydia trachomatis*

**[1]S Vesle, [2]M Nedeljkovic, [2]S Djukle, [1]T Mandic**

*[1]Institute of Dermatovenereology, Clinical Centre of Serbia, Belgrade, Yugoslavia, [2]Institute of Microbiology and Immunology, University of Belgrade, Yugoslavia*

A total of 1052 patients with nongonococcal uretheiris were included in this study. Detection of *Chlamydia trachomatis* from urethral secretions was made by using direct immunofluorescence test with FITC-labelled monoclonal antibodies (DIF). The indirect immunofluorescence test (IFA) was performed in order to determine the titre of IgM and IgG antichlamydial antibodies in patients' sera. *Chlamydia trachomatis* was detected in 545 (51.8%) out of 1052 patients with nongonococcal urethritis. Antichlamydial IgM antibodies (titre > 8) were detected in 436 (80.0%), and IgG antibodies (titre > 16), were detected in 473 (86.8%) out of 545 patients with *Chlamydia trachomatis*-positive nongonococcal urethritis. A good correlation between results obtained by the direct and indirect immunofluorescence tests was found (Rxy=0.71). Therefore, exact diagnosis of chlamydial urethral infection requires using both these methods (DIF and IFA). Priority should be given to DIF test, because it proves the existence of causative agent, while IFA test plays a complementary role and can be helpful in following up of the dynamics of chlamydial urethral infection.

---

### In Vitro Neutralization Of *Chlamydia pneumoniae*: Infectivity With Patient Sera and With Monoclonal Antibodies

**HM Freidank, M Wiedmann-Al-Ahmad**

*Institut fur Medizinische Mikrobiologie, Freiburg, Germany*

IgG and IgA antibodies to *Chlamydia pneumoniae* were determined with the micro-immunofluorescence (MIF) test according to Wang and Grayston in more than 2000 serum samples. 33 sera containing both IgG and IgA antibodies and 35 sera positive for IgG and negative for IgA antibodies to *C. pneumoniae* at different titers were selected for determination of their neutralizing activity following the method standardized for in vitro neutralization of *Chlamydia trachomatis* by Byrne et al. In addition, 21 sera negative for *C. pneumoniae* antibodies in the MIF test were examined as well as a monoclonal antibody against *C. pneumoniae* (RR-402, Washington Research Foundation) and a panel of new monoclonal antibodies produced against a purified 54 kDa protein of *C. pneumoniae*. Since studies on the in vitro neutralization of *C. trachomatis* showed that the results may depend on the cell line, the performance of different cell lines (HL, Baby hamster kidney, Buffalo Green Monkey kidney cells) was evaluated. 30 of the 35 patient sera with IgG antibodies and 31 of the 33 patient sera with both IgG and IgA antibodies showed neutralizing activity defined as >50% reduction in the inclusion count as compared to the untreated control. 20 of the 21 sera negative for *C. pneumoniae* antibodies in the MIF test did not neutralize infectivity, whereas both the monoclonal antibody RR-402 against *C. pneumoniae* and the monoclonal antibodies against the 54 kDa-protein of *C. pneumoniae* showed neutralization at a titer of $\geq$1:10000 and $\geq$1:80, respectively. The neutralizing ability of the sera correlated better with MIF IgG titers than with IgA titers.

---

### Chlamydia Infections in Andrologic Clinic: Laboratory Diagnosis

**I Gorpinchenko, L Dobrovolska, N Boyko**

*Institute of Urology and Nephrology, Ukraine*

**Objective:**To study the frequency of occurrence of *Chlamydia trachomatis* genitourinary infections in patients attending Andrologic clinic in Kiev Institute of Urology.

**Methods:** It were used method of light microscopy stained by Romanovsky-Gimze and immunofluorescent method with monoclonal antibody to detect *Chlamydia trachomatis* in urethra endothelial cells as well as indirect blood immunofluorescent method. 68 men were examined. All patients was divided into three groups: G1 - patients suffered from urethritis (23 men), G2 - patients with prostatitis (22 men) and G3 - men with urethroprostatitis - (23 men).

**Results:** Has been detected by three methods that genitourinary infections among examined men was caused by *Chlamydia trachomatis* in 39.7% of cases (27 men). In G1 *Chlamydia trachomatis* was defined in 34.8% (8 men), and in G2 - 36.4% (8 men). Among the patients of G3 *Chlamydia trachomatis* was observed more often (43.5% - 10 men). Mean frequency of occurrence of *Chlamydia trachomatis* for G3 was significantly higher than G1 and G2. Diagnostic values of light microscopy by Romanovsky-Gimze and immunofluorescent method with monoclonal antibody to *Chlamydia trachomatis* in urethra endothelial cells were 30% and 70% accordingly in comparative with indirect blood immunoflluorescent method. *Chlamydia trachomatis* was observed not only as monoinfection but also as mixed infection with other infection agents. The association with one kind of microorganisms was met more often (42.7%) than with two (36.7%) or especially three infection agents (18.6%).

**Conclusions:** These data demonstrate that *Chlamydia trachomatis* is detected as an infectious agent causing urethroprostatitis. The results show that indirect blood immunofluorescent method was the most available for diagnostics of *Chlamydia trachomatis* genitourinary infections.

### Circulating IgG Antibodies to the *Chlamydia trachomatis*, *Escherichia coli* And Human 60kD Heat Shock Proteins In Women

**S.S. Witkin[1], M. Askienazy-Elbhar[2], J. Henry-Suchet[3], J. Tort-Grumbach[3] K. Sarjdine[3]**

[1]*Dept of Obstetrics and Gynecology, Cornell University Medical College, New York, NY,* [2] *Laboratoire de Biologie Medicale Magenta, Paris, France and* [3]*Family Planning Center, Hospital Saint Louis, Paris, France*

The relation between circulating IgG antibodies to the *C. trachomatis* and *E. coli* 60 kD heat shock proteins (hsp60) and immunity to the corresponding human 60kD heat shock protein was examined. 103 serum samples from male and female subjects seen at the Hospital Saint Louis in Paris were tested. Purified recombinant *E. coli* hsp60 (GroEL) and human hsp60, both from StressGen (Victoria, BC, Canada), as well as synthetic peptides corresponding to unique (amino acids 151-162) and conserved (amino acids 260-271) epitopes of the *C. trachomatis* hsp6o were utilized to test for antibodies by ELISA. Antibodies to chlamydial hsp60 epitopes 151-162 and 260-271 were detected in 8 (7.8%) and 27 (26.2%) sera, respectively. Antibodies to the *E. coli* and human hsp60 were each identified in 11 (10.7%) samples. As expected the presence of antibodies to the two synthetic peptides were highly correlated (p=0.0002). Antibody to peptide 151-162 was detected in 26.9% of sera with, and in only 1.3% of sera without, antibody to peptide 260-271. Of major interest was the observation of the unique association between antibodies to the conserved chlamydial hsp60 peptide 260-271 and antibodies to human hsp60 (p=0.03). Antibodies to human hsp60 were present in 22.2% of women with, and in 6.6% of women without, antibodies to chlamydial hsp60 epitope 260-271. In marked contrast, there was no association between antibodies to chlamydial hsp60 epitope 151-162 or the *E. coli* hsp60 and antibodies to human hsp60. Antibodies to peptide 260-271 were more prevalent in women (35.1%) than in men (13.0%) (p=0.01). However, there was no difference in the prevalence of human hsp60 antibodies between men (10.9%) and women (10.5%). The data reinforce previous studies suggesting that immune sensitization to an epitope of the *C. trachomatis* hsp60 that is homologous to an epitope in the human hsp60 could lead to the induction of autoantibodies to the human hsp60 in some individuals. The relation between hsp60 antibodies and antibodies to *C. trachomatis* surface antigens and to clinical diagnosis is currently being evaluated.

## References

[PDF_00728] Brade L., Brunnemann H., Ernst M., Fu Y., Holst O., Kosma P., Näher H., Persson K., Brade H. (1994). Occurrence of antibodies against chlamydial lipopolysaccharide in human sera as measured by ELISA using an artificial glycoconjugate antigen.. FEMS Immunol Med Microbiol.

[PDF_00321] Buzoni-Gatel D., Guilloteau L., Bernard F., Bernard S., Chardès T., Rocca A. (1992). Protection against Chlamydia psittaci in mice conferred by Lyt-2+ T cells.. Immunology.

[PDF_00318] Buzoni-Gatel D., Rodolakis A., Plommet M. (1987). T cell mediated and humoral immunity in a mouse Chlamydia psittaci systemic infection.. Res Vet Sci.

[PDF_00615] Mahony J. B., Sellors J., Chernesky M. A. (1987). Detection of chlamydial inclusions in cell culture or biopsy tissue by alkaline phosphatase-anti-alkaline phosphatase staining.. J Clin Microbiol.

[PDF_00315] Rodolakis A. (1983). In vitro and in vivo properties of chemically induced temperature-sensitive mutants of Chlamydia psittaci var. ovis: screening in a murine model.. Infect Immun.

[PDF_00725] Yi Y., Zhong G., Brunham R. C. (1993). Continuous B-cell epitopes in Chlamydia trachomatis heat shock protein 60.. Infect Immun.

[PDF_00324] de Sa C., Souriau A., Bernard F., Salinas J., Rodolakis A. (1995). An oligomer of the major outer membrane protein of Chlamydia psittaci is recognized by monoclonal antibodies which protect mice from abortion.. Infect Immun.

